# Current Situation and Associated Factors of Withdrawing or Withholding Life Support to Patients in an Intensive Care Unit of Cancer Center in China

**DOI:** 10.1371/journal.pone.0098545

**Published:** 2014-05-28

**Authors:** Qingyu Zhao, Xiaodan Zhang, Yi Fang, Jian Gong, Baochun Gu, Gang Ma

**Affiliations:** 1 Intensive Care Unit, Sun Yat-sen University Cancer Center, Guangzhou, China; 2 Zhongshan School of Medicine, Sun Yat-sen University, Guangzhou, China; D'or Institute of Research and Education, Brazil

## Abstract

**Objectives:**

To investigate the current situation and analyze the associated factors of withdrawing or withholding life support in the intensive care unit (ICU) of our cancer center.

**Methods:**

Three hundred and twenty-two cancer patients in critical status were admitted to our ICU in 2010 and 2011. They were included in the study and were classified into two groups: withdrawing or withholding life support (WWLS), and full life support (FLS). Demographic information and clinical data were collected and compared between the two groups. Factors associated with withdrawing or withholding life support were analyzed with univariate and multivariate logistic regression analysis.

**Results:**

Eighty-two of the 322 cases (25.5% of all) made the decisions to withdraw or withhold life support. Emergency or critical condition at hospital admission, higher scores of Acute Physiology and Chronic Health Evaluation II (APACHE II) in 12 hours after ICU admission, financial difficulties and humanistic care requirements are important factors associated with withdrawing or withholding life support.

**Conclusions:**

Withdrawing or withholding life support is not uncommon in critically ill cancer patients in China. Characteristics and associated factors of the decision-making are related to the current medical system, medical resources and traditional culture of the country.

## Introduction

Cancer has already become one of the global leading causes of deaths, with annual death toll increasing by about 40% worldwide in the last 20 years [1]. It was predicted that annual death from cancer would rise from currently around 8 million to 13.2 million by 2030 [2]. The situation is worsening in developing countries, as the annual rate of new cases of cancer is expected to increase three times faster than the high-income countries [3]. For example, cancer has already replaced cardiovascular diseases and become the leading cause of deaths in Chinese adults [4].

In recent years, life expectancies of millions of cancer patients have been extended due to developments in anticancer medical treatments and critical life support. Nevertheless, for some patients at the final stages of cancer or with comorbidities such as multiple organ dysfunction and coma, supportive care in ICU can only sustain their lives for a short period of time. In the meantime, patients with consciousness have to suffer huge physical and psychological pain with extremely low quality of life while unconscious patients have to rely on monitoring devices and supportive medications. In these cases, withdrawing or withholding life support, as another option, is often considered by physicians, patients and their families. Instructions and guidelines have been released in the Western countries concerning this issue [5]–[6]. As a result, incidence of such decisions was increasing during these years [7]. In clinical practices in China, the decisions to withdraw or withhold treatments are usually made by patients and/or their families with or without implications of physicians. However, due to the complicated unsolved ethics problems and the lack of a related legal system in China, no standard or recommended procedure of withdrawing or withholding treatments can be accessed and few reports about these decisions for the critically ill patients in ICU can be found. Presently in the clinical work, we could find that some patients receive undue treatments and take excessive medical resources at the final stage of life, while some others at early stage of cancer give up critical care for various reasons. It is therefore essential to investigate the situation and associated factors of withdrawing or withholding life support (WWLS) among the critically ill cancer patients in the ICU. The present retrospective study has been carried out to demonstrate these clinical patterns in the ICU of Sun Yat-sen University Cancer Center, which is one of the largest cancer centers in China and that has been treating cancer patients mainly from southern and central China.

## Materials and Methods

The study was approved by the Clinical Research Ethics Committee of Sun Yat-sen University Cancer Center. Informed consent was not obtained due to the retrospective and observational nature of the study.

### 1. Participants

At the time of the study, the ICU of Sun Yat-sen University Cancer Center was an 18-bed department with 9 physicians (3 chief, 4 attending and 2 resident physicians) and 45 nurses. Other medical staff may include clinical graduate students of the University, doctors from other departments of the Cancer Center for rotational training, and doctors from other hospitals' ICUs for further training. Cancer patients who were critically ill or just underwent major surgeries were admitted for intensive care and treatment. Families were allowed to visit the patients twice a day, from 11:00 am to 12:00 pm and 07:00 pm to 08:00 pm, one by one alternately. Inventories of the patients' daily medical expenses were given to their families each day and families could consult with the doctors in charge about the situation, treatments, prognosis, costs and other issues concerning them.

The study was conducted from January 1, 2010 to December 31, 2011. Patients admitted to our ICU for intensive supportive care during this period were included. Definitions used to classify the decisions to remove or terminate life support were listed as follows: Withdrawal of life support (WDLS): The cessation and removal of ongoing intensive life support therapy (e.g. mechanical ventilation, dialysis, vasoactive agents, and immunological support such as large doses of gamma globulin and thymosin which were expensive and had to be self-paid in the ICU) with the explicit intent not to substitute an equivalent, alternative treatment. Withholding of life support (WHLS): The decision not to start or increase a medically appropriate or potential beneficial life support therapy in the ICU.

Participants were classified into two groups according to the decision, withdrawing or withholding life support (WWLS) group and full life support (FLS) group. In WWLS cases, a specific document for withdrawing or withholding life support was signed by the patients' families when a consensus decision was made. All patients were followed up until death in the ICU or discharge from the ICU.

### 2. Measurement and data collection

Acute Physiology and Chronic Health Evaluation (APACHE) II was taken to assess the temporal pathophysiological status. The highest score in 12 hours after admission to ICU was noted as APACHEII_0_ and the highest score during ICU stay was noted as APACHEII_1_.

Demographic information, basic health information and clinical data during hospitalization, and especially the data during ICU stay, were collected retrospectively by two independent researchers according to the medical records. Besides the data which could be noted accurately during treatment, we included two additional items for analysis, namely financial difficulties and humanistic requirements. Financial difficulties were considered by ICU physicians comprehensively according to the concern about medical costs, place of residence, source of medical costs and employment of patients and their families. Generally speaking, if the patients' medical expenses were not supported by health care insurance of government or company, and if their monthly salary or pension was lower than their daily expenses in the ICU, they would run into economic distress soon. Humanistic care requirements were divided into two categories: one was the requirement to limit traumatic or invasive rescue measures such as external chest compression and tracheotomy to avoid further suffering or injury, the other was the requirement to follow the traditional end-of-life customs and take the patient back home.

### 3. Statistical analyses

Student t- test or Wilcoxon rank-sum test was used for the continuous data, expressed as Mean ± Standard Deviation (SD) and Median (Interquartile range, IQR). Chi-square or Fisher's exact test was used for the categorical variables. Factors associated with withdrawing or withholding life support were analyzed with univariate and multivariate logistic regression. Significant factors in univariate analysis were included in multivariate models. A two-tailed p-value <0.05 was considered statistically significant. All data were analyzed using the statistical software SPSS 17.0 for Windows.

## Results

### 1. Clinical data of patients

Three hundred and twenty-two patients were included initially and 23 of them were admitted to the ICU more than once during our study. For patients with multiple admissions, the last admission was adopted for analysis. Data of 322 cases were collected in total: 82 (25.5%) cases in WWLS group and 240 (74.5%) cases in FLS group. In FLS cases, physicians also considered to withhold life support for some patients (24/240) according to their physical status and once implied their families who insisted to continue. Some patients (16/240) themselves showed the intention to withdraw life support, but their families chose to continue after consulting with the physicians. Most (92.7%) of the decisions were made by patients' families without intervention of physicians, while only a few (7.3%) were affected by the professional advices from the ICU physicians, surgeons or oncologists. Nurses were not involved in the process. Documents for withdrawing or withholding life support were signed in 80 cases. In the other 2 cases, the families refused to sign the document due to domestic disputes.

With respect to demographic and basic health information, no significant difference was shown between two groups in age, sex, employment, source of medical expense, primary tumor, cancer stage and chronic diseases (See Table 1). However, more patients in WWLS group were in financial difficulties, in emergency or critically condition at hospital admission, and received non-surgical treatment before ICU admission. Patients or their families in WWLS group expressed humanistic care requirements more often.

**Table 1:** Demographic and basic health information of patients.

Characteristics	Frequency (Rate)		p
	WWLS (n = 82)	FLS (n = 240)	
Gender			0.600
Male	64 (78%)	177 (74%)	
Female	18 (22%)	63 (26%)	
Age (year)	56.6±16.1	58.1±16.8	0.459
Employment			0.795
Rural work	6 (7.3%)	24 (10.0%)	
Urban work	19 (23.2%)	49 (20.4%)	
Government work	7 (8.5%)	16 (6.7%)	
No employment	50 (61.0%)	151 (62.9%)	
Status at hospital admission			0.012
Emergency or critical condition	15 (18.3%)	20 (8.3%)	
General	67 (81.7%)	220 (91.7%)	
Source of medical expense			0.332
Public	1 (1.2%)	5 (2.1%)	
Health insurance	9 (11.0%)	41 (17.1%)	
Individual	72 (87.8%)	194 (80.8%)	
Financial difficulties	25 (39.5%)	1 (0.4%)	<0.001
Humanistic care requirements	36 (43.9%)	14 (5.8%)	<0.001
Primary tumor			0.063
Respiratory	19 (23.2%)	50 (20.8%)	
Alimentary	34 (41.5%)	100 (41.7%)	
Urogenital	5 (6.1%)	24 (10.0%)	
Neural	6 (7.3%)	3 (1.2%)	
Lymphoma	9 (11.0%)	30 (12.5%)	
Head and neck	5 (6.1%)	9 (3.8%)	
Others	4 (4.9%)	24 (10.0%)	
Cancer stage (TNM staging^a^)			0.120
I	4 (4.9%)	24 (10.0%)	
II	20 (24.4%)	69 (28.8%)	
III	16 (19.5%)	58 (24.2%)	
IV	42 (51.2%)	89 (37.0%)	
Basic chronic diseases			
Hypertension	12 (14.6%)	25 (10.4%)	0.301
Diabetes	6 (7.3%)	16 (6.7%)	0.840
Cardiac disease	2 (2.4%)	10 (4.2%)	0.707
Treatment before ICU admission			0.006
Surgery	28 (34.1%)	131 (54.6%)	
Chemotherapy	22 (26.8%)	50 (20.8%)	
Radiotherapy	2 (2.4%)	12 (5.0%)	
Intervention	6 (7.3%)	9 (3.8%)	
Supportive care	24 (29.3%)	38 (15.8%)	

a The TNM Classification of Malignant Tumors (TNM).

Concerning with duration and expenses of treatments, time of continuous renal replacement treatment (CRRT) and daily average medical costs of WWLS group were significantly longer or higher, while duration of hospitalization, hospitalized times and duration of ICU stay were lower compared with FLS group. No significant difference was demonstrated in duration of disease, total medical costs and time of mechanical ventilation (See Table 2).

**Table 2:** Comparison of clinical data between two groups.

	WWLS (n = 82)	FLS (n = 240)	p
Duration of hospitalization (day)	15(7–33)	25(16–42)	<0.001
	22.4±20.6	34.2±37.8	
Hospitalized times	2(1–4)	1(1–2)	0.011
	3.2±3.6	3.0±4.6	
Total course of disease (month)	3(2–8)	2(2–5)	0.119
	11.9±27.0	9.6±25.3	
Total medical costs (RMB)	88491.2(32169.3–179814.7)	111207.0(59881.1–164106.0)	0.073
	128499.6±134319.0	141477.3±139543.3	
Daily Average medical costs (RMB)	5618.9(3783.5–8710.7)	4197.0(3155.2–5624.6)	<0.001
	6367.4±3397.9	4749.0±2418.0	
Duration of mechanical ventilation (hour)	30.5(5.0–146.5)	43.0(2.0–120.0)	0.875
	155.4±295.4	132.8±432.4	
Duration of CRRT (hour)	0.0(0.0–0.0)	0.0(0.0–0.0)	0.015
	15.8±56.3	9.0±40.2	
Duration of ICU stay (day)	3.5(1.0–9.2)	6(3–10)	0.008
	8.8±13.0	9.3±18.1	

Data were expressed as Median (IQR) and Mean ± SD.

Indicated by APACHE II scores, health conditions of patients in WWLS group deteriorated apparently. Some treatments such as CRRT and vasopressors were more often given to WWLS patients. No significant difference was shown in comorbidities at ICU admission between two groups (See Table 3).

**Table 3:** Status of patients and treatments during ICU stay.

		WWLS (n = 82)	FLS (n = 240)	p
Health evaluation				
APACHEII_0_	Median (IQR)	15 (12–20)	12 (10–15)	<0.001
	Mean ± SD	17.1±7.5	13.4±5.7	
APACHEII_1_	Median (IQR)	22 (15–32)	13 (11–19)	<0.001
	Mean ± SD	23.3±9.2	16.8±9.0	
Comorbidities at ICU admission				
Cardiac arrest		11 (13.4%)	17 (7.1%)	0.079
Hemorrhage		12 (14.6%)	26 (10.8%)	0.357
Sepsis		13 (15.9%)	39 (16.2%)	0.933
Treatment in ICU				
Mechanical ventilation		68 (82.9%)	185 (77.1%)	0.266
CRRT		16 (19.5%)	22 (9.2%)	0.012
Transfusion		17 (20.7%)	37 (15.4%)	0.266
Vasopressors		40 (48.8%)	82 (34.2%)	0.019
Pathological status in ICU				
Coma		38 (46.3%)	56 (23.3%)	<0.001
MODS/MOF		48 (58.5%)	60 (25.0%)	<0.001
Death		18 (22.0%)	42 (17.5%)	0.371

### 2. Patterns of WWLS cases

The decision to withdraw or withhold life support was made in 82 cases in our study, out of which 20 cases (24.4%) withdrew all active treatments, and the other 62 cases (75.6%) withheld some of the life support measures, mostly external chest compression and/or electrical defibrillation. Among these WWLS cases, 27 (32.9%) were reluctant to make the decisions due to unsolved problems such as financial difficulties, family disputes, foster relationship and limited local medical resources. The other 55 (67.1%) were prone to make the decisions. 36 cases (43.9%) expressed humanistic care requirements and 25 cases (30.5%) were in financial difficulties. There were a total of 18 deaths (22%) in the ICU after the decisions to withdraw or withhold life support, which was 30% of all deaths in the ICU. The other 64 patients (78%) discharged from hospital and returned home or to local hospitals.

### 3. Factors associated with withdrawing or withholding life support

All cases in WWLS group made their decisions to withdraw or withhold life support before leaving ICU. Considering the lack of a specific time in FLS group corresponding to the time of decision in WWLS group, we ruled out several incomparable factors from analysis, including duration of mechanical ventilation and CRRT, duration of hospitalization, medical costs and death at ICU discharge. The other possible associated factors were all involved in statistical analysis. Results of univariate analysis revealed associated factors including emergency or critical condition at hospital admission, total course of illness >3 months, treatment before ICU admission, APACHEII_0_>15, APACHEII_1_>22, CRRT in ICU, vasopressors in ICU, coma in ICU, MODS/MOF in ICU, financial difficulties and humanistic care requirements (See Table S1). Multivariate analysis revealed associated factors including emergency or critical condition at hospital admission, APACHEII_0_>15, financial difficulties and humanistic care requirements (See Table 4).

**Table 4:** Multivariate logistic regression analysis of withdrawing or withhold life support.

Factors	p	OR	95%CI for OR	
			Lower	Upper
Financial difficulties	<0.001	110.654	14.032	872.624
Humanistic care requirements	<0.001	12.708	5.909	27.330
Emergency or critical condition at hospital admission	0.010	3.285	1.335	8.085
APACHEII_0_>15	0.042	2.035	1.026	4.034

OR: Odds Ratio. CI: Confidence Interval.

## Discussion

For cancer patients suffering from immune deficiency and malnutrition, anti-cancer treatments can lead to life-threatening complications, such as organ dysfunction or failure. Life support therapies in intensive care unit are essential for these cases. However, the use of monitoring devices and large amounts of medications may cause huge physical sufferings and economic burden. As a result, decisions to limit treatments are usually made in special cases.

Withholding or withdrawing life support, which challenges the old idea that lives should be sustained at any cost, is now considered rationally, taking into account many factors including patient and their families' request, patient's quality of life, prognosis and the principle of fair distribution of medical resources. Incidence of withdrawing or withholding treatments varies between different ICUs and countries [7]–[10]. Rate of limiting life support in patients dying in ICUs varies from 21% to 96% in America [8] and ranges from 20% [9] to 71.4% [10] in Europe. A study of an ICU in Hong Kong showed that limitation of life support occurred in 58.8% of all deaths in the ICU [11]. In our study, the rate was 30%, much lower than many general hospitals. But the rate of making decisions to limit life support therapies in all ICU patients was 25.5%, higher than other reports revealing the rate less than 10% [12]–[14]. This may be explained by the specificity of cancer patients. Without symptoms, cancer cases can hardly be detected at early stages and the prognosis is often unknown. Therefore, most cancers are still not curable currently and anticancer treatment is a long-drawn, expensive process. When compared with patients with other chronic diseases, cancer patients usually have to experience much more psychological stress and economic burden in addition to physical sufferings. It was reported that critically ill patients with malignancy or ultimately fatal underlying diseases were more likely to have their life-sustaining therapy withheld or withdrawn than those without malignancy or fatal diseases [15]–[16]. A study of a specialized cancer center in Jordan demonstrated a high proportion (48.6%) of adult cancer patients making decisions to withhold or withdraw life support measures [17], which was even higher than the result of our center.

In accordance with some domestic researches [18]–[19], family financial condition and humanistic care requirements were important factors in correlation with WWLS decisions in our study, which was related to the medical system, medical resources and traditional cultures in our country. In contrast to studies in other countries which showed that economic cost played no role in end-of-life decisions [20], financial problem can be one of the core considerations for our patients and their families. Since the medical system is not well-developed yet, a considerable proportion of the Chinese population is not covered by health insurance. Even with the health insurance provided by government, most patients still have to pay a relatively high proportion (30–80%) of their cost [21]. Furthermore, for patients with malignant or critical diseases, their medical costs are usually much higher than the others and certain amounts of expensive medications or treatment fees for these diseases could not be covered by health insurance. Therefore, the huge financial cost of critical care is a leading obstacle for many patients to proceed. However, since it is inappropriate to investigate the accurate financial condition of others in Chinese culture, we were unable to get the accurate financial data such as income, deposit or estate of our patients. Comprehensive evaluation regarding financial status was done by ICU physicians instead, thus the accuracy of the outcome might be affected. We noticed that 48.8% of WWLS patients were in early or middle stage of cancer (below stage IV) which could be effectively treated, indicating that nonclinical factors including financial difficulties played important roles in decision-making. Sometimes medical disputes or even violence can occur because of high medical costs. To reduce possible dissatisfaction of the family caused by the treatment and medical costs, we provided lists of daily medical expenses and communicated with the family during visiting hours each day. End-of-life traditional customs, classified as humanistic care, are also common reasons for WWLS decisions. Being a country with a long history, China has a wide variety of traditional customs about death, the core of which is body ground burial. Although cremation has been mandatory and already been put into practice in cities like Guangzhou, burial is still the most common way to deal with the dead bodies in rural areas. For some patients in critical condition, families sometimes choose to withdraw treatments and return home or to local hospitals, in order to take the patient back to their hometown before their last breath which is considered as existence of the spirit and to prepare for the traditional burial soon after their death. Besides, some people with religious beliefs think it unnatural and inauspicious to die in hospitals. They prefer to let life end at home peacefully. As the old saying goes, “Falling leaves return to their roots”, this has been the prevalent view of death of the Chinese people since ancient times. Studies in Taiwan demonstrated that nearly 90% of the terminal cancer patients preferred to die at home [22] and 25% of patients dying in surgical ICU returned home to die [23].

Apart from financial difficulties and humanistic care requirements, status at hospital admission and high APACHE II scores after ICU admission were also factors associated with WWLS decisions, which indicated that severe physical conditions had important impacts on treatment decision. Other studies stated similar findings [14], [19], [24] and indicated that poor prognosis [8], [25], [26] was one of the main reasons for withdrawing treatment.

There is no standard WWLS procedure in China so far. Also, despite of the fact that some guidelines and instructions have been published, practices of end-of-life decisions vary substantially in different countries and regions [27]. The process of decisions making commonly seen in our country greatly differs from the one in European countries and is similar to the one in the United States. According to studies in European countries [13], [14], [20], [25], despite of the data variance, most end-of-life decisions (>50%) were made or initiated by the medical staff, especially physicians, with or without participation of patients and their families. In the United States, in accordance with state laws and hospital policies, the decisions were usually made by patients or their surrogates in consultation with physicians [28]–[29]. In Chinese culture, familial relationships and rights are sometimes stressed more than an individual's rights, as being revealed in a study in Hong Kong [11]. Therefore, in most medical cases, especially for critically ill cancer patients, family members instead of the patients are the very ones who physicians directly communicate with, in order to avoid evoking overwhelming psychological stress. This may explain why most of the decisions were made by families without intervention of physicians in our study.

According to some researches [10], [26], [28], [30], patients' age was one of the main reasons for treatment withdrawal or limitation. In our study, nevertheless, age was not an important factor associated with the decisions, possibly be owing to the traditional Chinese culture, in which the old are respected as supreme and children grow up in the culture of filial duty. Supporting the well-being and saving the lives of parents in their later years are regarded as the unavoidable responsibilities of the children. Even China's law at present declares the children's obligation to support the parents while living and to give them proper burial after death.

Patients who receive surgery are usually at early or middle stage of cancer. In comparison, those who receive chemotherapy, radiotherapy, intervention or supportive care, are generally at a later stage of cancer. Our results showed that more patients were at lower cancer stage and were postoperative in FLS group than in WWLS group. The difference in hospitalization times indicated that WWLS patients received more anticancer treatments before, but none of these are factors attributable to treatment decisions.

The median duration of ICU stay for WWLS patients was 3.5 days, indicating that half of the patients and their families made WWLS decisions in less than 4 days after admission to ICU. The decisions directly led to a decrease in duration of hospitalization and medical costs. The results revealed a general decrease in medical resource waste and economic burden by withdrawing or withholding life support.

In conclusion, our study reveals that withdrawing or withholding life support is not uncommon in critically ill cancer patients in China. Emergency or critical condition, financial difficulties and humanistic care requirements are important factors associated with WWLS decisions. Nonclinical factors rather than clinical conditions of patients play significant roles in some of the cases. Retrospective study in only one center may confine the value of our conclusion, therefore we sincerely appeal for more multicenter investigations to help set up guidelines and to promote the systems of palliative care in China.

## Supporting Information

Table S1 **Univariate logistic regression analysis of withdrawing or withholding critical care.**
(DOCX)
