# Supplementary material for: Current Situation and Associated Factors of Withdrawing or Withholding Life Support to Patients in an Intensive Care Unit of Cancer Center in China
Source: PLoS One. 2014 May 28;9(5):e98545. doi: 10.1371/journal.pone.0098545 (PMC4037202; doi:10.1371/journal.pone.0098545)
Supplement: Table S1 — Univariate logistic regression analysis of withdrawing or withholding critical care. (DOCX) [file pone.0098545.s001.docx]

Table. Univariate logistic regression analysis of withdrawing or withholding critical care.

| Factors | p | OR | 95%CI for OR | |
| --- | --- | --- | --- | --- |
|  |  |  | Lower | Upper |
| Age | 0.478 | 0.995 |  |  |
| Gender | 0.439 | 0.790 |  |  |
| Cardiac arrest at ICU admission | 0.084 | 2.032 |  |  |
| Sepsis at ICU admission | 0.864 | 0.942 |  |  |
| Hemorrhage at ICU admission | 0.359 | 1.411 |  |  |
| With diabetes | 0.840 | 1.105 |  |  |
| With hypertension | 0.359 | 1.411 |  |  |
| With cardiac disease | 0.481 | 0.575 |  |  |
| APACHEⅡ_0_ >15 | <0.001 | 2.832 | 1.667 | 4.812 |
| APACHEⅡ_1_ >22 | <0.001 | 3.280 | 1.934 | 5.565 |
| Mechanical ventilation in ICU | 0.267 | 1.444 |  |  |
| CRRT in ICU | 0.014 | 2.402 | 1.192 | 4.839 |
| Coma in ICU | <0.001 | 2.905 | 1.713 | 4.927 |
| Vasopressors in ICU | 0.019 | 1.835 | 1.104 | 3.052 |
| MODS/MOF in ICU | <0.001 | 4.235 | 2.499 | 7.178 |
| Transfusion in ICU | 0.268 | 1.435 |  |  |
| Treatment before ICU admission (Compared with radiotherapy) | 0.007 |  |  |  |
| Surgery | 0.045 | 0.203 | 0.043 | 0.962 |
| Chemotherapy | 0.002 | 0.355 | 0.185 | 0.683 |
| Intervention | 0.420 | 0.745 |  |  |
| Supportive care | 0.892 | 1.083 |  |  |
| Employment (Compared with jobless) | 0.815 |  |  |  |
| Rural work | 0.572 | 0.762 |  |  |
| Urban work | 0.384 | 0.571 |  |  |
| Government work | 0.789 | 0.869 |  |  |
| Source of medical expenses (Compared with public) | 0.361 |  |  |  |
| Health insurance | 0.576 | 0.539 |  |  |
| Individual | 0.182 | 0.591 |  |  |
| Cancer stage (Compared with stage Ⅳ) | 0.127 |  |  |  |
| Ⅰ | 0.069 | 0.353 |  |  |
| Ⅱ | 0.122 | 0.614 |  |  |
| Ⅲ | 0.113 | 0.585 |  |  |
| Primary tumor (Compared with other tumors) | 0.125 |  |  |  |
| Respiratory | 0.121 | 0.300 |  |  |
| Alimentary | 0.397 | 0.606 |  |  |
| Urogenital | 0.187 | 0.375 |  |  |
| Neural | 0.155 | 3.600 |  |  |
| Lymphoma | 0.540 | 0.684 |  |  |
| Head and neck | 0.389 | 0.599 |  |  |
| Hospitalized times >2 | 0.05 | 1.717 | 1.000 | 2.948 |
| Total duration of disease >3 months | 0.016 | 1.881 | 1.124 | 3.147 |
| Emergency or critical condition at admission | 0.010 | 2.604 | 1.255 | 5.404 |
| Financial difficulties | <0.001 | 104.825 | 13.912 | 789.846 |
| Humanistic care requirements | <0.001 | 11.739 | 5.945 | 23.188 |

OR: Odds Ratio. CI: Confidence Interval.
